# Rapid Clearance of microRNA-371a-3p: A Longitudinal Study of Serum Levels in Patients with Localized Testicular Germ Cell Tumors

**DOI:** 10.1016/j.euros.2026.09.008

**Published:** 2026-10-10

**Authors:** Klaus-Peter Dieckmann, Tabea Niemzok, Jan Diesend, Mattis Franke, Markus Klemke, Alexander Harms, Christian Wülfing, Nina Wagener, Gazanfer Belge

**Affiliations:** a Asklepios Klinik Altona, Department of Urology, Hamburg, Germany; b University of Bremen, Faculty of Biology and Chemistry, Bremen, Germany; c mir|detect GmbH, Bremerhaven, Germany; d MVZ Hanse Histologikum, Hamburg, Germany

**Keywords:** Germ cell tumor, Tumor marker, Decay of marker levels, Half-life, Time to normalization, microRNA-371a-3p

## Abstract

**Background and objective:**

Rapid clearance after treatment is a cornerstone feature of valuable tumor markers. microRNA-371a-3p (M371) has much promise to become a useful biomarker of germ cell tumors (GCTs). However, little is known about its velocity of decay. This study aimed to systematically analyze the kinetics of decline of M371 serum levels.

**Methods:**

A total of 53 patients with clinical stage (CS) I GCT had measurements of M371 levels before orchiectomy and five measurements postoperatively, four within the first 48 h, and one after 2 wk. The commercial M371 test was used to measure serum levels with quantitative polymerase chain reaction. Postoperative levels were statistically compared with the preoperative level. The half-life (H/L) and the absolute time to normalization (TTN) were calculated by linear interpolation. Possible associations with clinical factors were evaluated.

**Key findings and limitations:**

The median H/L of M371 was 3.8 h (interquartile range [IQR] 2.9–6.2). We found no statistical evidence that H/L was associated with the clinical factors evaluated. The absolute median TTN was 33.8 h (IQR 6.5–261.6), which was associated with the baseline value of M371. Limitations may result from baseline blood sampling several days before surgery.

**Conclusions and clinical implications:**

M371 levels in patients with CS I GCT decline with a H/L of 3.8 h postoperatively, which is much more rapid than in traditional markers. The absolute TTN of M371 is associated with the baseline value and exhibits interindividual variability. Overall, the rapid kinetic profile of M371 is another valuable feature of the novel biomarker of testicular GCTs.

## Take Home Message

Serum levels of microRNA-371a-3p drop very rapidly with a half-life of 3.8 h after treatment. A few cases require a longer time to normalize. The kinetic profile of this biomarker allows rapid recognition of treatment response and offers prospects for future therapeutic strategies.

## Graphical abstract

**Figure:**
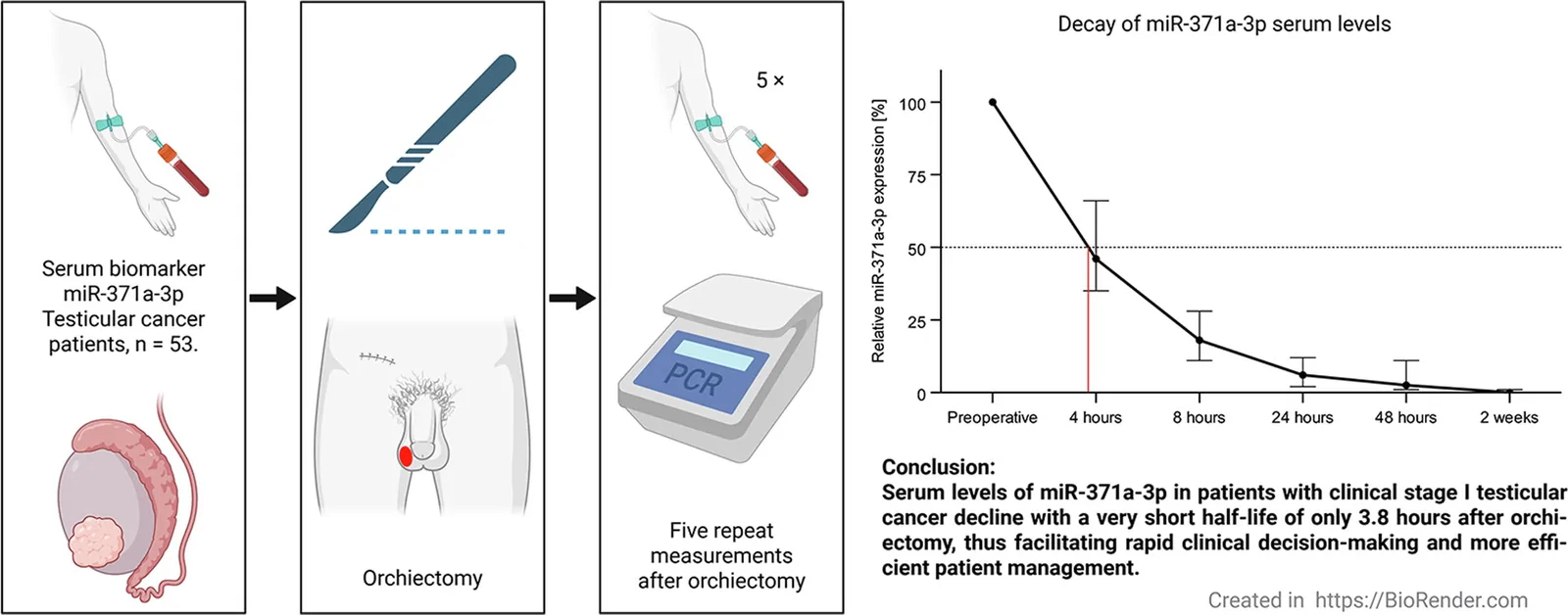

ADVANCING PRACTICE **What does this study add?**

Serum levels of microRNA-371a-3p (M371) decline very rapidly and much faster than those of traditional markers after treatment. This result may provide a rationale for future studies evaluating clinical pathways using the rapid clearance of M371.

**Clinical Relevance**

53 patients with clinical stage I testicular germ cell tumors, serum microRNA-371a-3p declined rapidly after orchiectomy, with an estimated median half-life of 3.8 hours. However, approximately 10% remained above the assay threshold at 2 weeks, highlighting variability in normalization. These findings may inform postoperative sampling strategies, but persistent elevation cannot yet reliably establish residual disease or guide treatment. Prospective validation is required before incorporating these kinetics into clinical decision-making. Associate Editor: Maria Carmen Mir.

**Patient Summary**

Serum levels of microRNA-371a-3p (M371) are considered a promising biomarker of testicular germ cell tumors. This study revealed a much more rapid decline of M371 after treatment than that of traditional markers. Further research will show whether this feature can facilitate a more rapid assessment in clinical settings than is currently possible.

## 1 Introduction

Serum levels of microRNA-371a-3p (M371) fulfill almost all the conditions of an ideal tumor marker of testicular germ cell tumors (GCTs) [1], [2], [3] as outlined by Lange and Winfield [4] in 1987. In the primary diagnosis of GCTs, M371 revealed a sensitivity of 90% and a specificity of >90% [5], [6], [7]. The marker is not expressed in other malignancies [8], [9]. Relapses in clinical stage (CS) I GCTs can be detected with a sensitivity and specificity of 100% and 96.3%, respectively [10], [11], [12]. M371 serum levels correlate with tumor burden and response to treatment [13], [14], [15], [16], [17].

However, one of the key features has received little attention so far: the rapid clearance of the marker substance from serum after treatment [4]. To date, only one small pilot study has systematically assessed the velocity of decay and reported a serum half-life (H/L) of M371 <12 h after orchiectomy. That study also found that the time to normalization (TTN), which is the interval from surgery until the M371 level declines below the cutoff value, was associated with tumor size [18]. However, the level of evidence was low because of the small patient sample (*n* = 24) and low frequency of M371 measurements. Support came from a Norwegian study that reported a clearance of M371 of <24 h in four patients [13]. By contrast, a recent study from the USA observed patients exhibiting elevated M371 values even 3 wk after orchiectomy [19].

Knowledge about the velocity of decay of a tumor marker is essential for clinical decision-making, particularly when assessing the response to treatment. In GCTs, clinical management is impeded by the very long H/L of alpha-fetoprotein (AFP) of 5–7 d and the fairly long H/L of beta human chorionic gonadotropin (bHCG) of ∼36 h [20], [21], [22]. This investigation aimed to analyze the decay of serum M371 levels in a descriptive way by examining a sizeable cohort of patients with GCT with a rather high frequency of serum sampling after treatment. This is a biomarker kinetics study that does not evaluate whether M371-guided decisions can improve clinical management. Secondary end points were possible associations between the velocity of decay and clinical characteristics.

## 2 Patients and methods

### 2.1 Study population, clinical methods

In a prospective study, consecutive patients undergoing inguinal orchiectomy for testicular tumor in Asklepios Klinik Altona, Hamburg, during 2024–2025, meeting the following criteria were enrolled: (1) histologically proven testicular GCT, (2) CS I according to the European Association of Urology (EAU) [23], [24], and (3) aged >18 yr. Exclusively CS I cases were recruited because only in this setting can a tumor-free status be assumed after treatment. Serum samples were collected before orchiectomy and repeatedly at five postoperative checkpoints: 4 h, 8 h, 24 h, 2 d, and 2 wk. Because of clinical organizational problems, actual sampling times usually differed from planned time points by ∼10%. Planned time points were used for statistical analysis. The following clinical data were recorded for each patient: histology (seminoma; nonseminoma), patient age (yr), tumor size (mm), CS Ia or CS Ib according to EAU [23], [24], body mass index (BMI; kg/m^2^), and preoperative (baseline) M371 serum level. Serum values of AFP and bHCG were measured before orchiectomy and postoperatively on day 1 and after 2 wk.

Control subjects were male patients aged 18–60 yr who were treated for nonmalignant diseases at Asklepios Klinik Altona during 2024–2025; each provided one blood sample. The study followed the Reporting Recommendations for Tumor Marker Prognostic Studies guidelines regarding marker kinetic studies. Standards relating to evaluation of diagnostic performance and clinical marker utility were not applicable. All patients gave written informed consent for participation. Ethics approval was provided by the ethics committee of Ärztekammer Bremen (#301/11, May 30, 2011 and #301/17, September 21, 2017). All study activities were conducted in conformity with the Declaration of Helsinki of the World Medical Association, amended by the 75th General Assembly in October 2024.

### 2.2 Laboratory methods

Aliquots of serum samples were stored at –80°C until final analysis. Measurement of M371 serum levels was performed as detailed earlier [5], [9]. In short, a real-time quantitative polymerase chain reaction was performed using the In Vitro Diagnostic Medical Devices Regulation–certified M371-Test (mir|detect, Bremerhaven, Germany) with miR-30b-5p as the endogenous control. M371 serum levels were reported as relative quantities (RQ values) according to the ΔΔCt method [25]. The cutoff value was defined as RQ = 10.0 in accordance with the specifications of the manufacturer of the test (mir|detect GmbH, Bremerhaven, Germany).

Serum levels of AFP and bHCG were determined according to institutional standard operating procedures as reported earlier [26].

### 2.3 Statistical methods

Clinical data and laboratory results were initially stored in a commercially available database (Microsoft Excel version 2511; Microsoft Corp., Redmond, WA, USA). Final statistical analysis was performed after transferring all data into SPSS (version 31.0.1.0; IBM Corp., Armonk, NY, USA, RRID: SCR_002865).

Receiver operating characteristic curve analysis was performed using preoperative measurement results (preRQ) of patients with GCT and serum levels of controls to confirm that the sensitivity and specificity of the test in the present patient cohort did not differ from previous results and to ensure the appropriateness of the predefined cutoff value using Youden index analysis.

Median M371 serum levels with corresponding interquartile ranges (IQRs) were calculated for all measurement points. The preRQ values were statistically compared with the RQ values of the controls using the Mann–Whitney U test. Comparisons between median M371 values at consecutive time points were performed using the Wilcoxon signed-rank test.

The H/L was defined as the period from surgery to the time point at which M371 serum levels were predicted to reach 50% of baseline. The H/L of M371 was calculated for each patient by normalizing baseline levels to 100% and applying linear interpolation to the two measurements bracketing a 50% decline. TTN was defined as the interval from surgery to the time point at which the M371 level was predicted to reach the cutoff value (details regarding the calculation of H/L and TTN in supplementary text documents).

To evaluate possible associations of H/L or TTN with clinical factors, Spearman correlation analysis was performed using the continuous variables age, BMI, tumor size, and baseline RQ value. Categorical parameters were compared as follows: histology (seminoma vs nonseminoma) and CS (CS Ia vs CS Ib).

## 3 Results

A total of 69 patients with testicular tumor had originally been included. Two cases with non-GCT histology and 14 with CS >1 were excluded from analysis. Thus, the study population comprised 106 participants: 53 patients with GCT (32 with seminoma and 21 with nonseminoma) and 53 controls. Their clinical characteristics are summarized in Table 1. A total of 337 measurements of M371 serum levels were performed, including 53 in controls, 53 in patients with GCT preoperatively, 51 at 4 h postoperatively, 48 at 8 h, 53 at 24 h, 30 at 2 d, and 47 at 2 wk after orchiectomy.

**Table 1:** Clinical characteristics of study participants

Subgroups and characteristics	Results
Patients with GCT, *n*	53
Age of GCT patients (yr), median; IQR	34; 29–47
Tumor size (mm), median; IQR	32; 16–52
BMI (kg/m^2^), median; IQR	25.5; 23.8–27.8
Seminoma, *n* (% of GCTs)	32 (60)
Age (yr), median; IQR	35; 30–49
Tumor size (mm), median; IQR	28; 16–52
BMI (kg/m^2^) (median; IQR)	25.7; 23.6–28.8
Nonseminoma, *n* (% of GCTs)	21 (40)
Age (yr), median; IQR	32; 28–43
Tumor size (mm), median; IQR	42; 16–48
BMI (kg/m^2^), median; IQR	25.0; 24.3–27.4
Control subjects, *n*	53
Age (yr), median; IQR	43; 34–56
Hydroceles, *n*	19
Spermatoceles, *n*	16
Epididymitis, *n*	14
Varicocele, *n*	4

GCT = germ cell tumor; IQR = interquartile range; BMI = body mass index.

The calculated cutoff for the serum M371 level (RQ = 8.78; Supplementary Table 1) was very close to the predefined cutoff. All further analyses used RQ = 10.0 as the cutoff.

The median preoperative M371 level in patients with GCT was preRQ = 124.8 (IQR 42.7–429.7). Subgroup results are listed in Supplementary Table 2.

Postoperatively, the median M371 level dropped to 46% of the baseline level at the 4-h time point. The computed H/L was 3.8 h (IQR 2.9–6.2; 95% confidence interval [CI] 3.2–5.7). The rapid decline is illustrated in Figure 1 and Supplementary Figure 1; statistical details are provided in Supplementary Table 3.

**Fig. 1:**
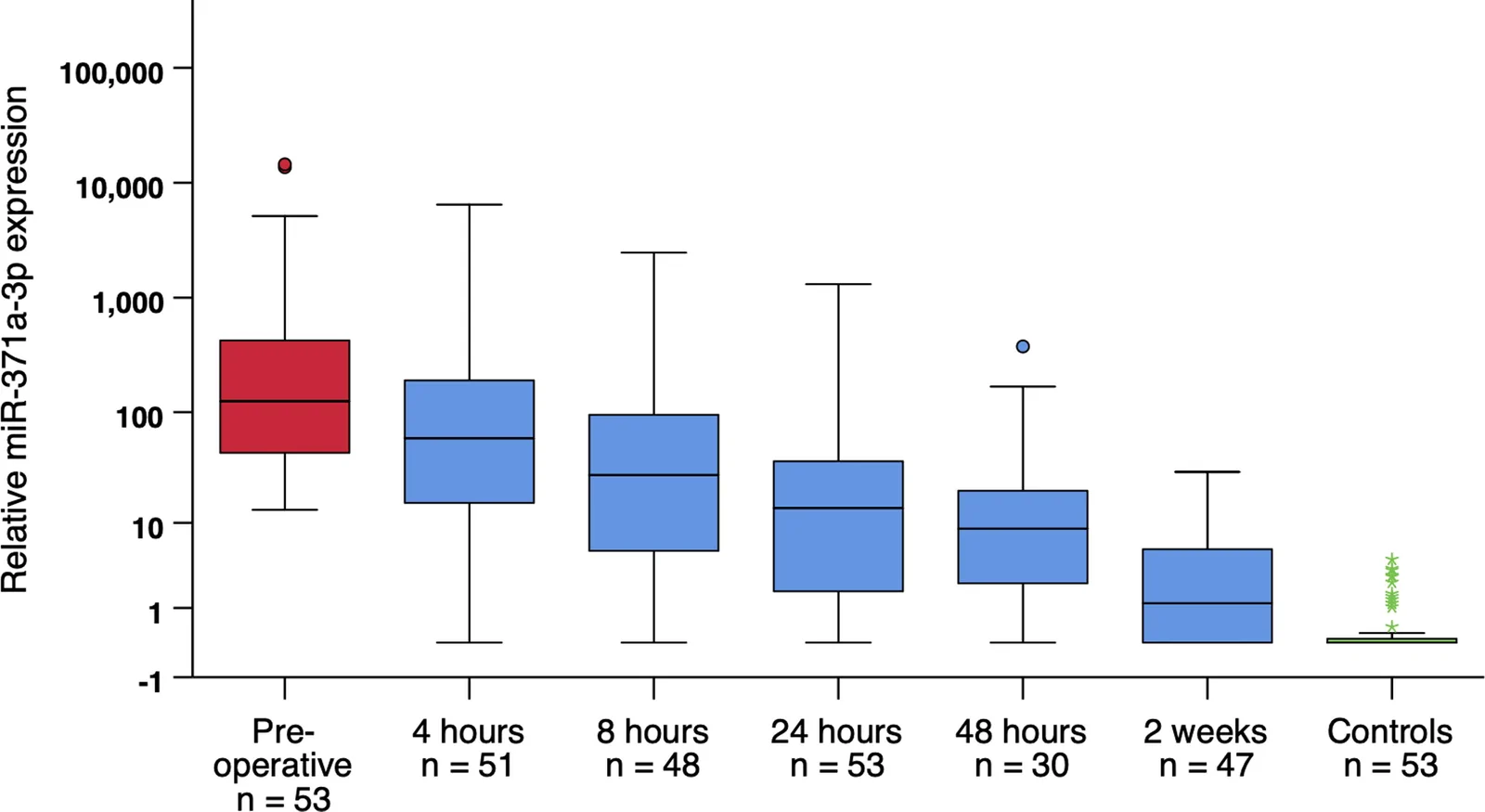
Median serum levels of microRNA-371a-3p at consecutive postoperative measurement points. Box-and-whisker plot showing the median microRNA-371a-3p serum levels at consecutive postoperative measurement points. There is a rapid and constant decline in serum levels. Red box: preoperative baseline level. Each box displays the first quartile, median, and third quartile of relative quantity values. Whiskers are defined as the largest and smallest observed values that fall within 1.5 times the interquartile range above the third quartile and below the first quartile, respectively. The y-axis is displayed on a logarithmic scale. Dots represent outliers. The x-axis is categorical and not proportional to elapsed time. Statistical details are provided in Supplementary Table 3.

Fig. 2 outlines the relative postoperative decline of M371 serum levels in individual patients, illustrating the overall rapid decline. However, some individual cases were different from the common pattern. Six patients showed slight increases or a plateau during the first 4 h after surgery. At the 24-h checkpoint, two still had serum levels of >50% of baseline.

**Fig. 2:**
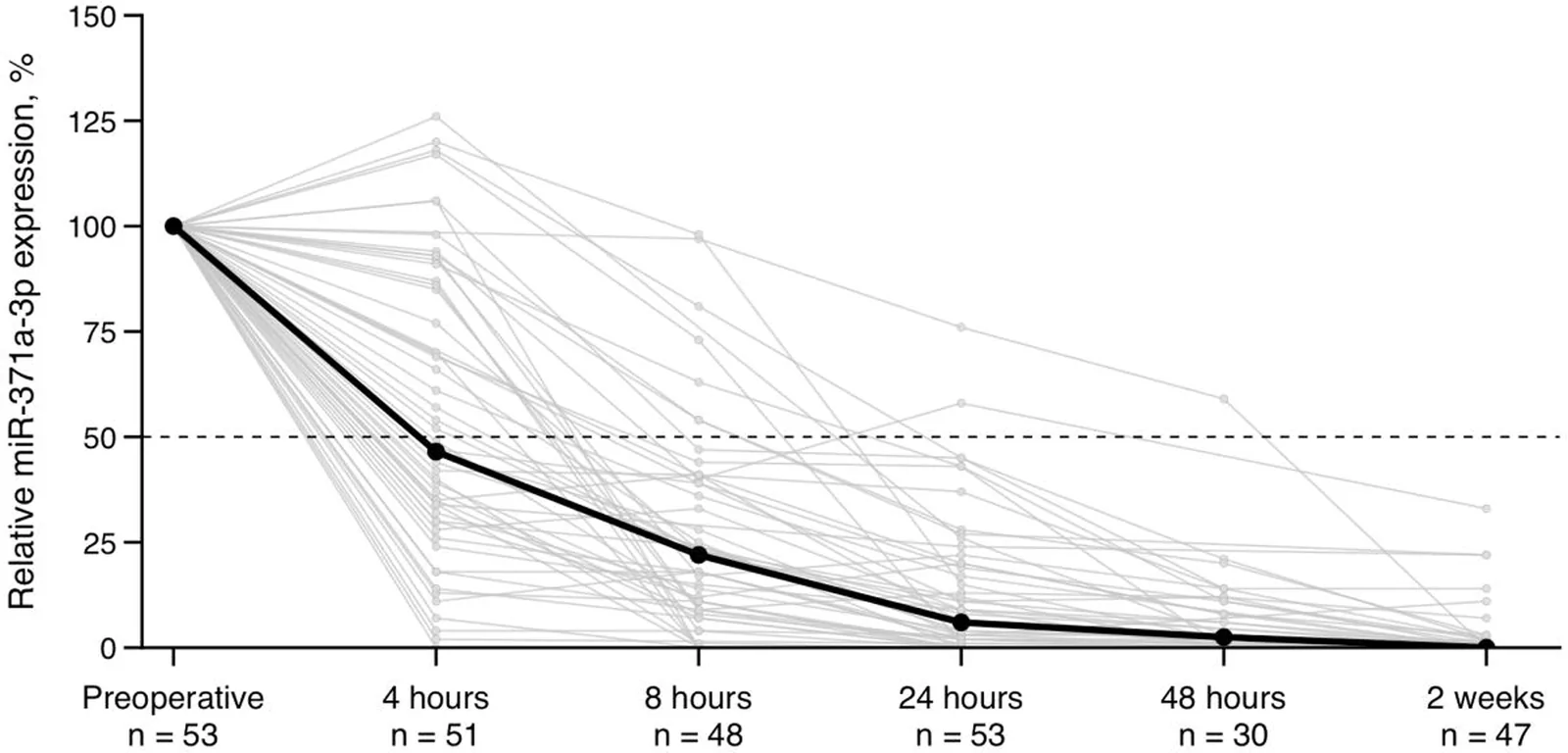
Relative decline of microRNA-371a-3p serum levels after orchiectomy in individual cases. Line graph representing the relative decline (ie, percentage of the baseline value) of microRNA-371a-3p levels in each individual patient during the 2 wk after orchiectomy. The bold line represents the median trajectory. The x-axis is categorical and not proportional to elapsed time. More than 50% of the cases dropped to <50% of the baseline level within 4 h after surgery. Six cases showed a slight increase or a plateau phase initially. After 24 h, only 2 cases still had serum levels of >50% of the preoperative level.

Clinical re-evaluation of the patients with unusually slow postoperative decline revealed that two of them had their baseline values measured at 13 and 18 d, respectively, before orchiectomy (details in Supplementary Tables 4 and 5). Two others had very low preRQ values (15.4 and 13.3).

Regarding the absolute postoperative decline in M371 levels (Supplementary Fig. 2), the median TTN was 33.8 h (IQR 6.5–261.6; 95% CI 19.8–142.5). Five patients (9.4%) had M371 serum levels above the threshold after 2 wk, three of whom had very high baseline levels (preRQ >1000), one developed a relapse 18 mo after orchiectomy, and one other was aged 87 yr (Supplementary Table 4).

Spearman correlation analysis did not reveal any significant association of H/L with clinical factors (Table 2). By contrast, TTN showed significant positive correlations with baseline RQ, tumor size, CS, and age, with the latter to a lesser extent (Table 2). These results are derived from exploratory analyses with multiple testing.

**Table 2:** Associations of serum H/L of microRNA-371a-3p and TTN with clinical factors. Results of Spearman correlation analysis

	H/L		TTN	
Clinical factor	Spearman correlation coefficient (*r_s_*)	*p* value	Spearman correlation coefficient (*r_s_*)	*p* value
BMI	–0.107	0.445	0.150	0.284
Age	–0.047	0.740	0.276	0.045
Tumor size	–0.041	0.771	0.639	<0.001
Baseline RQ	–0.019	0.894	0.699	<0.001
Clinical stage (CS1a vs CS1b)	0.042	0.765	0.379	0.005
Histology (SE vs NS)	–0.018	0.900	–0.035	0.802

All results are derived from exploratory analyses with multiple testing.

H/L = half-life; TTN = time to normalization; BMI = body mass index; RQ = relative quantity; CS = clinical stage; SE = seminoma; NS = nonseminoma.

Only 11 and 21 patients with GCT had elevated preoperative AFP and bHCG levels, respectively. The computed H/L was 18.9 h (IQR 17.0–23.7) and 188.7 h (IQR 134.8–236.0) for bHCG and for AFP, respectively. At 2 wk postoperatively, bHCG and AFP were still elevated in 19% and 36%, respectively, and normalized subsequently.

## 4 Discussion

The central result of this study is the exceptionally short M371 H/L of 3.8 h in patients with CS I disease following orchiectomy. Importantly, 9.4% of the patients require >2 wk to decline below the cutoff.

### 4.1 H/L

The M371 H/L of 3.8 h is markedly shorter than the half-lives of bHCG (36 h), AFP (5–7 d), and lactate dehydrogenase (∼24 h) [20], [21]. This finding is consistent with an H/L of <12 h found in pilot investigations [13], [18]. However, this analysis used a much more granular assessment than previous studies by using four repeated measurements during the first 48 h, in contrast to daily postoperative examinations used in former studies. Thus, the H/L of 3.8 h found herein probably represents a robust and valuable result that is supported by the narrow IQR (2.9–6.2 h).

### 4.2 Association of H/L with clinical factors

The precise mechanisms governing the biological clearance of circulating M371 are not known. However, given that M371 is undetectable in urine [27] and its decay rate is independent of renal function [19], it must be hypothesized that the clearance is likely mediated by blood-borne degradation, presumably via specific ribonuclease activity.

Accordingly, there was no statistically significant evidence of association of the relative rate of decay with any of the clinical factors analyzed. This finding is consistent with the concept that H/L reflects the relative postoperative decay rate of M371 and is therefore expected to be largely independent of the initial biomarker concentration or tumor burden.

An apparently slow decline resulted from a statistical issue in two cases; both had very low baseline levels (RQ 13.3 and 15.46). Their percentage decline appeared to be rather low, although absolute values had dropped below the threshold.

In contrast to the relative velocity of decay, the total absolute TTN of M371 serum levels is probably stochastically related to the number of circulating M371 molecules on one side and to the amount of degrading enzymes on the other side. Accordingly, Spearman correlation analysis revealed significant correlations of TTN with baseline M371 levels, tumor size, and CS. These findings are in line with expectations because patients with higher initial M371 levels require several H/L periods to decline below the cutoff. Likewise, tumor size and CS are associated with TTN, as both factors have previously been associated with higher preoperative M371 expression.

### 4.3 Interindividual variability of the TTN

The absolute TTN varies widely (IQR 6.5–261.6 h), with ∼10% of the patients not dropping below the cutoff within 2 wk postoperatively.

Very high baseline M371 levels (RQ 1632–2279) are a common feature of the slowly declining cases. However, in a model calculation with a given median H/L of 3.8 h, the drop below cutoff should have been accomplished after 7–8 H/L periods with an expected TTN of ∼36–48 h in patients with such baseline levels. In five cases, the observed TTN was much longer than 2 wk.

One reason for persistently elevated M371 levels after orchiectomy may be occult disease in regional lymph nodes, indicating a high risk of relapse [28], [29]. Accordingly, clinical re-evaluation disclosed that one of the patients relapsed 18 mo later.

Another patient with a very long TTN was aged 87 yr and also had an excessively elevated baseline M371 level (RQ = 2279.7). One hypothesis is that age-dependent regression of enzyme metabolism could have delayed the decline in serum levels. This postulation is supported by the significant correlation between age and TTN found herein. However, this issue needs further consideration because only very few older patients were included.

In two cases with postoperatively increasing or plateauing serum levels, the preoperative measurement had been conducted about 2 wk before surgery. Conceivably, tumor progression during the waiting period might have caused an increase in serum levels, making the first postoperative miR level appear higher than baseline. Preoperative M371 measurements were usually done on the day of the GCT diagnosis. The waiting time for orchiectomy averages 5–10 d, but a few patients had longer intervals. Thus, tumor progression during the preoperative waiting period could account for some of the slow declines.

In all, M371 levels may decline more slowly in some cases, and the underlying biological reasons are not fully understood. Consequently, caregivers of patients with GCT should be aware of slow decline in M371 expression in isolated cases, particularly in those with very high baseline levels.

### 4.4 Prospects arising from the short H/L

The exceptionally short H/L of M371 is likely to represent another unique characteristic of this biomarker, complementing its high sensitivity and specificity as well as its expression across both major histologic subgroups of GCTs. Although formal clinical evidence is currently lacking, the rapid clearance kinetics of M371 may prove valuable in clinical situations for which existing management strategies remain suboptimal. One such scenario is the persistent elevation of conventional serum tumor markers in patients with nonseminomatous GCT following orchiectomy. Also, this finding may provide a rationale for prospective, controlled studies to determine whether the remarkably rapid clearance of M371 can be incorporated into diagnostic and therapeutic algorithms for GCTs.

### 4.5 Limitations

Confounding effects might have resulted from the fact that baseline M371 levels had usually been measured several days before surgery. In fact, only 18 patients had their blood draws on the day of orchiectomy, whereas 23, 10, and 2 patients were examined earlier, at intervals of 1–10 d preoperatively, 10–14 d, and >2 wk, respectively. Thus, serum levels may have increased during the waiting period, possibly affecting the calculated H/L. Another limitation might result from missing measurement values at several postoperative checkpoints. The 23 missing values at the 48-h checkpoint were attributable to hospital administrative constraints, not to clinical problems. Specifically, the missing values at this time point could have biased the calculation of the median TTN.

A minor limitation might result from not accounting for the histologic subtypes of nonseminoma with their known wide variance of M371 expression [5], [30].

With respect to the statistical analysis, individual H/L were calculated using linear interpolation analysis, assuming a linear decline of serum levels between two measurement points, although the true biological course of miR levels probably does not exactly follow that assumption.

The rapid decay of M371 found herein specifically relates to localized disease (CS I). It is rational to assume a similarly rapid clearance in response to chemotherapy in advanced cases, as indicated previously [5], [28], [31], [32]. However, M371 levels will probably decline less rapidly in metastatic cases because chemotherapy eradicates tumor masses gradually in contrast to orchiectomy in CS I cases, in which the entire mass is immediately removed by surgery.

## 5 Conclusions

M371 serum levels in patients with CS I GCT decline with an H/L of 3.8 h after orchiectomy, which is markedly shorter than the H/Ls of the traditional markers. About 10% of the cases require a markedly longer absolute TTN. Overall, the findings support further prospective validation, ideally with baseline sampling on the day of surgery. If validated, this study results could also provide a rationale for evaluating clinical utility end points associated with the rapid kinetic profile of M371.

  ***Author contributions:*** Gazanfer Belge had full access to all the data in the study and takes responsibility for the integrity of the data and the accuracy of the data analysis.

  *Study concept and design*: Belge, Dieckmann, Niemzok.

*Acquisition of data*: Franke, Niemzok, Dieckmann, Wagener.

*Analysis and interpretation of data*: Belge, Niemzok, Dieckmann, Franke, Wagener, Harms, Diesend.

*Drafting of the manuscript*: Dieckmann, Belge, Niemzok, Klemke, Franke.

*Critical revision of the manuscript for important intellectual content*: All authors.

*Statistical analysis:* Niemzok, Belge, Diesend, Klemke.

*Obtaining funding*: None.

*Administrative, technical, or material support*: Belge, Niemzok.

*Supervision*: Wülfing, Wagener.

*Other*: Histological examinations: Harms.

  ***Financial disclosures:*** Gazanfer Belge certifies that all conflicts of interest, including specific financial interests and relationships and affiliations relevant to the subject matter or materials discussed in the manuscript (eg, employment/affiliation, grants or funding, consultancies, honoraria, stock ownership or options, expert testimony, royalties, or patents filed, received, or pending), are the following: Klaus-Peter Dieckmann and Gazanfer Belge each possess a 3.7% ownership share in mir|detect GmbH, Bremerhaven. Tabea Niemzok, Jan Diesend, and Markus Klemke are employees of mir|detect GmbH, Bremerhaven. All other authors have nothing to disclose.

  ***Funding/Support and role of the sponsor:*** Support came from Institut für Klinische Forschung, Semmelweiss Universität, Campus Hamburg. This institution collects and administers donations from private individuals who wish to express their gratitude (https://ikf.hamburq/).

  ***Acknowledgments:*** The authors are grateful to all patients included in the present study for their confidence and willingness to participate. The technical assistance provided by the staff of the Medilys laboratory at Asklepios Klinik Altona is gratefully acknowledged. All assistant physicians of the Department of Urology, Asklepios Klinik Altona, provided valuable aid in collecting blood samples during duty hours.

  ***Data availability:*** The data are available from the corresponding author upon reasonable request.

## Appendix A Supplementary data

Supplementary Data 1 Supplementary tables, figures and text documents.
