# Supplementary material for: Rapid Clearance of microRNA-371a-3p: A Longitudinal Study of Serum Levels in Patients with Localized Testicular Germ Cell Tumors
Source: Eur Urol Open Sci. 2026 Oct 10;94:8–14. doi: 10.1016/j.euros.2026.09.008 (PMC13678096; doi:10.1016/j.euros.2026.09.008)
Supplement: Supplementary file 1 [file mmc1.docx]

**Supplementary files**

**Supplementary Table 1**

Performance characteristics of the M371 test in the present cohort

| **Parameter** | **Result** | **95% CI** |
| --- | --- | --- |
| AUC | 1.000 | 0.93 - 1.00 |
| Sensitivity | 100 % | 93 - 100% |
| Specificity | 100 % | 93 - 100% |
| Cut-Off (RQ) | 10.0 (8.78)* | - |

Performance characteristics were calculated with receiver operating characteristics (ROC) analysis by using RQ=10.0 as predefined cutoff. *RQ=8.78 is the ROC/Youden-derived theoretical cutoff value that was not employed in the present analysis. Youden index analysis served to confirm that the predefined cutoff value is very close to the formal calculated one and can safely be used in the present analysis.

95% CI confidence intervals; AU area under the curve; RQ relative quantity.

**Supplementary Table 2**

Median preoperative M371 serum levels in various subgroups

|  | **(n)** | **Median RQ value (IQR)** |
| --- | --- | --- |
| All GCT | 53 | 124.8 (42.8 – 229.7) |
| Seminoma | 32 | 120.4 (43.1 – 278.6) |
| Nonseminoma | 21 | 141.2 (38.1 – 1597.8) |
| CS1a* | 24 | 59.5 (22.4 – 124.3) |
| CS1b* | 29 | 308.9 (111.1 – 1697.1) |

*The difference between CS1a and CS1b is statistically significant

CS clinical stage; IQR interquartile range; GCT germ cell tumour

**Supplementary Table 3**

Postoperative decrease of M371 serum levels: statistical comparisons of median RQ values determined at various time-points

| **Time-point** | **Eligible**  **(n)** | **Compared to** | **Eligible (n)** | **p-value** | **test** |
| --- | --- | --- | --- | --- | --- |
| baseline | 53 | controls | 53 | <0,001 | Mann Whitney U |
| baseline | 53 | 4 h | 51 | <0,001 | Wilcoxon signed rank test |
| 4 h | 51 | 8 h | 48 | <0,001 | Wilcoxon signed rank test |
| 8h | 48 | 24 h | 53 | <0,001 | Wilcoxon signed rank test |
| 24 h | 53 | 48 h | 30 | <0,001 | Wilcoxon signed rank test |
| 48 h | 30 | 2 weeks | 47 | <0,001 | Wilcoxon signed rank test |

This table shows comparisons of median M371 levels (RQ-Values) determined at various postoperative time-points; baseline: median preoperative RQ-value; n number of patients eligible; h hours; p-values are derived from exploratory analyses

**Supplementary Table 4a**

Clinical characteristics of 6 patients with transiently increasing M371 serum levels after orchiectomy

| **Patient #** | **Baseline**  **RQ** | **Time-point of measurement** | **RQ after 4 h**  **(%)** | **Age**  **(years)** | **histology** | **Tumour size** | **CS1a / CS1b** |
| --- | --- | --- | --- | --- | --- | --- | --- |
| 1 | 1770.57 | 18 days before surgery | 2082.24  (118%) | 45 | SE | 49 mm | CS1b |
| 2 | 166.57 | 13 days before surgery | 194.60  (117%) | 56 | SE | 62 mm | CS1b |
| 3 | 141.15 | day of surgery | 169.55  (120%) | 32 | NS  (90% EC, 10% TE) | 17 mm | CS1b |
| 4 | 5135.52 | 7 days before surgery | 6462.10  (126%) | 34 | NS  (100% EC) | 58 mm | CS1b |
| 5 | 124.78 | day of surgery | 131.91  (106%) | 41 | NS  (80% TE, 10% YST, 8% EC, 2% SE) | 49 mm | CS1a |
| 6 | 13.99 | day of surgery | 14.83  (106%) | 27 | NS (40% EC, 40% YST, 15% SE, 5% TE) | 11 mm | CS1a |

RQ relative quantity, i.e. the M371 serum level measured; baseline measurement before orchiectomy; SE seminoma; NS nonseminoma; TE teratoma; YST yolk sac tumour; EC embryonal carcinoma; TS tumour size; CS clinical stage. All percentages are in relation to the preoperative M371 level

**Supplementary Table 4b:**

Clinical characteristics of patients with slow decline (>50% of baseline after 1 day)

| **Patient #** | **Baseline RQ** | **4 h RQ** | **8 h RQ** | **24 h RQ** | **48 h RQ** | **2 weeks RQ** | **histology** | **Tumour size** | **CS1a/ CS1b** |
| --- | --- | --- | --- | --- | --- | --- | --- | --- | --- |
| 7 | 15.46 (100%) | - | 15.06 (97%) | 11.68 (76%) | 9.11 (59%) | 0 (0%) | SE | 11 mm | CS1a |
| 8 | 13.31 (100%) | 12.49 (94%) | 5.3 (40%) | 7.71 (58%) | - | 4.43 (33%) | SE | 7 mm | CS1a |

This table lists the M371 serum levels (RQ values) measured at baseline and at the following time-points. The last three columns provide clinical details of the patients

RQ relative quantity, i.e. M371 serum level; SE seminoma; CS clinical stage. All percentages are in relation to the preoperative M371 level

**Supplementary Table 5**

Patients with M371 serum levels still above cut-off at the 2 weeks’ time-point postoperatively

| **Patient #** | **RQ Preope-rative** | **RQ after 2 weeks** | **RQ**  **later** | **Indivi-dual H/L (h)** | **Tumour size (mm)** | **BMI**  **(kg/m^2^)** | **Age (years)** | **Histo-logy** |
| --- | --- | --- | --- | --- | --- | --- | --- | --- |
| 9 | 1770.6 | 29.52 | n.a. | 21.85 | 49 | 26.9 | 45 | SE |
| 10 | 1632.5 | 11.48 | 0.0  (9 weeks) | 3,58 | 52 | 33.3 | 46 | SE |
| 11 | 2279.7 | 21.38 | 0.161  (7 weeks) | 4,55 | 42 | 29.3 | 87 | NS |
| 12 | 140.4 | 19.47 | n.a. | 3,44 | 72 | 40.9 | 32 | SE |
| 13 | 71.6 | 16 | n.a. *relapse | 3,02 | 26 | 26.4 | 49 | SE |

RQ relative quantity (M371 serum level); n.a. not available; H/L half life; h hours; SE seminoma; NS nonseminoma; * this patients later relapsed

**Supplementary Figure 1**

**Relative decline of microRNA-371a-3p serum levels in the entire patient group**

Line graph shows postoperative decline of median serum levels at postoperative measurement points as percentage of baseline serum level. Error bars denote 95% Cis. The x-axis is categorical and not proportional to elapsed time.

This graph illustrates that the median M371 serum level is less than 50% of baseline already at the 4 hours check-point.

**Supplementary Figure 2**

**Absolute decline of serum microRNA-371a-3p serum levels after orchiectomy in individual cases**

Line-graph representing the course of microRNA-371a-p serum level in each individual patient during the two weeks postoperative observation period. Dashed line represents cut-off. Bold overlay line represents the median course of all cases.

The x-axis is categorical and not proportional to elapsed time.

More than 60% of cases, drop below cut-off value within 48 hours after surgery. Five cases (9.4%) were still above the threshold two weeks postoperatively.

**Supplementary text document #1**

**Calculation of half-lives and time to normalisation**

Half-life (H/L) and time to normalization (TTN) were calculated individually for each patient by linear interpolation between the two consecutive sampling time points surrounding the respective target value. For two adjacent measurements $\left( t_{1} , y_{1} \right)$ and $\left( t_{2} , y_{2} \right)$, the slope was calculated as $m=\frac{y_{2}-y_{1}}{t_{2}-t_{1}}$and the intercept as $b=y_{1}-mt_{1}.$The resulting linear function was therefore $y(t)=mt+b.$

The time point at which the target value was reached was obtained by rearranging the equation: $t=\frac{y_{\mathrm{target}}-b}{m},$ where $y_{\mathrm{target}}$ corresponds to either 50% of the individual preoperative baseline (half-life) or the predefined cut-off value of RQ = 10 (TTN).

Planned postoperative sampling times (4 h, 8 h, 24 h, 48 h and 14 days after orchiectomy) were used for all calculations. The identical predefined calculation algorithm was applied to all patients. No case-specific adjustments or exceptions were made. Planned postoperative sampling times were used to ensure a standardized calculation across all patients and to avoid introducing additional variability caused by minor deviations in the actual sampling times.

Missing values: Missing measurements were not imputed. Calculations were performed using the available measurements only. If one of the two consecutive sampling points surrounding the target value was missing, interpolation was performed using the nearest available measurement before and after the target value.

Plateauing values: Plateauing values did not require special handling, as interpolation was always performed using the two consecutive measurements surrounding the respective target value, irrespective of the overall shape of the decay curve.

Increasing values: Temporarily increasing values did not require special handling. The calculation was based on the first pair of consecutive measurements surrounding the predefined target value (50% of baseline or the cut-off value).

Values already below the cut-off: If the first postoperative measurement was already below the cut-off, TTN was calculated by interpolation between the preoperative baseline measurement and the first postoperative measurement.

Patients not reaching the cut-off: Patients whose serum miR-371a-3p levels did not decrease below the cut-off value within the 14-day observation period were identified separately. As the exact TTN could not be determined within the observation period, these patients were assigned a value of 500 hours solely for ranking purposes in the calculation of the cohort median, but not for correlation analysis. Because the median depends only on the rank order of the observations, the exact value assigned (provided it exceeded the maximum observed TTN) did not affect the reported median.

The following example illustrates the calculation procedure using Patient 7 from Supplementary Table 3b. Patient 7 (seminoma, tumour size 11 mm, CS1a) had a preoperative miR-371a-3p level of RQ = 15.46. The corresponding postoperative measurements were RQ = 15.06 after 8 h, RQ = 11.68 after 24 h, RQ = 9.11 after 48 h and RQ = 0 after two weeks.

**Example calculation of TTN**

The predefined cut-off (RQ = 10) lies between the measurements obtained after 24 h (RQ = 11.68) and 48 h (RQ = 9.11).

The slope is therefore $m=\frac{9.11-11.68}{48-24}=-0.1071$

and the intercept is $b=11.68-\left( -0.1071\times24 \right)=14.25$.

Thus, the corresponding linear equation is $y\left( t \right)=-0.1071t+14.25$. The calculated time point at which the cut-off is reached is $t=\frac{10-14.25}{-0.1071}=39.7\text{ }h$. Accordingly, the TTN for Patient 7 was 39.7 hours.

The same calculation procedure was subsequently applied for the determination of half-life.

**Example calculation of half-life**

The individual half-life corresponds to 50% of the preoperative baseline:

Baseline: $RQ_{\mathrm{baseline}}=15.46$

Half of baseline: $RQ_{50\%}=0.5\times15.46=7.73$

This value lies between the measurements obtained after 48 h (RQ = 9.11) and two weeks (336 h; RQ = 0).

The slope is $m=\frac{0-9.11}{336-48}=-0.0316$

and the intercept is $b=9.11-\left( -0.0316\times48 \right)=10.63$.

Thus, $y(t)=-0.0316t+10.63$. The calculated time point at which 50% of the baseline value is reached is $t=\frac{7.73-10.63}{-0.0316}=91.7\text{ }h$.

Accordingly, the calculated half-life for Patient 7 was 91.7 hours.

**Supplementary text document #2**

**Use of linear interpolation for calculation of H/L and TTN**

Although the biological clearance of circulating miR-371a-3p is generally expected to follow an exponential or log-linear decay pattern, linear interpolation was chosen in the present study as a pragmatic approximation. Importantly, not the entire postoperative decline was modeled linearly. Instead, interpolation was performed only between the two adjacent sampling points surrounding the respective target value (50% of the baseline value or the predefined cut-off). Owing to the short intervals between postoperative sampling time points, particularly during the early postoperative phase (4, 8, 24 and 48 hours), the difference between linear and log-linear interpolation within these intervals is expected to be rather small. Since interpolation was restricted to two adjacent sampling points surrounding the respective target value rather than the entire postoperative decay curve, the approximation error introduced by assuming linear instead of exponential decay is expected to be minimal.

For illustrating the comparison of interpolation methods, the median postoperative course of relative miR-371a-3p expression was used as an example. The median preoperative value was normalized to 100%, whereas the median relative expression level four hours after orchiectomy was 46%. Since the target value for half-life determination corresponds to 50% of the baseline value, it lies between the measurements obtained at 0 and 4 hours and therefore allows a direct comparison of linear and log-linear interpolation.

Linear interpolation: $t=0+\frac{50-100}{46-100}\times(4-0)=3.70\text{ }h$

Log-linear interpolation: $t=0+\frac{ln(50)-ln(100)}{ln(46)-ln(100)}\times(4-0)=3.57\text{ }h$

The two approaches differed by only 0.13 hours (approximately 8 minutes). This example demonstrates that, owing to the short intervals between early postoperative sampling time points, the numerical difference between linear and log-linear interpolation is minimal. Therefore, linear interpolation represents an appropriate approximation for estimating half-life and TTN within the sampling intervals used in the present study.
